# Antiretroviral therapy improves survival among TB-HIV co-infected patients who have CD4+ T-cell count above 350cells/mm^3^

**DOI:** 10.1186/s12879-016-1916-1

**Published:** 2016-10-17

**Authors:** Simon Mutembo, Jane N. Mutanga, Kebby Musokotwane, Lutangu Alisheke, Christopher C. Whalen

**Affiliations:** 1 Ministry of Health, Southern Provincial Medical Office, Choma, Zambia; 2 Department of Epidemiology and Biostatistics, College of Public Health, University of Georgia, Health Sciences Campus, 101 Buck Road, Athens, Georgia 30602 USA

**Keywords:** Antiretroviral therapy, HIV, Tuberculosis, Survival, CD4+ T-cell count > 350cells/mm^3^

## Abstract

**Background:**

Co-infection with *Mycobacterium tuberculosis* remains a leading cause of morbidity and mortality among HIV infected individuals especially in developing countries. Early initiation of cART in these patients when CD4+ T cell count is less than 200cells/mm^3^ has reduced disease progression and mortality. However for patients with higher CD4+ T cell counts greater than 350cells/mm^3^ evidence is conflicting. In this study we seek to evaluate the effectiveness of cART in reducing mortality among TB-HIV co-infected patients with CD4 + T cells above 350cells/mm^3^ at the time of TB diagnosis.

**Method:**

In a retrospective cohort study we analyzed 337 HIV-TB co-infected patients with CD4+ T cells above 350cells/mm^3^ at baseline who were diagnosed between 2006 and 2012 in the southern province of Zambia. The primary outcome was all-cause mortality. We estimated the effect of cART by comparing survival according to cART and controlling for differential loss to follow-up.

**Results:**

Of the 257 patients on cART, 22 died (9 %) and 20 (8 %) were lost to follow-up; of 80 patients not on cART, 20 died (25 %) and 19 (24 %) were lost to follow-up. Patients treated with cART had better survival compared to those not treated (*P* < 0 · 0001, log-rank test). In a proportional hazard regression adjusting for Cotrimoxazole, the risk of death was reduced by 78 % with cART (95 % CI: 0 · 47, 0 · 91). In a propensity score analysis, the effect of cART was still beneficial.

**Conclusion:**

In patients with HIV-associated TB and CD4+ T cells above 350cells/mm^3^, treatment with cART reduced mortality for up to 4 years as compared to no cART and was associated with better retention in care.

**Electronic supplementary material:**

The online version of this article (doi:10.1186/s12879-016-1916-1) contains supplementary material, which is available to authorized users.

## Background

Co-infection with *Mycobacterium tuberculosis* remains a leading cause of morbidity and mortality among Human Immunodeficiency Virus (HIV) infected individuals especially in developing countries [1, 2]. HIV-infected individuals are not only at high risk of developing Tuberculosis (TB), but also at increased risk for severe forms of the disease, recurrent disease and high mortality [3, 4]. The use of continuation Anti-Retroviral Treatment (cART) has reduced disease progression and death among patients with HIV infection, but the optimal time for initiating therapy is uncertain for patients co-infected with TB [5, 6]. For TB patients with CD4+ T cell counts lower than 200 cells/ mm^3^, the effects of cART on survival are convincing, [7–10] but for patients with CD4+ T cell counts above 350 cells/mm^3^, the effects of cART on survival are conflicting [11].

Some studies have concluded that early initiation of cART during treatment for TB improves survival among patients, [7–10] whereas other studies have not found this same benefit [11]. Based on the best available evidence and expert opinion, the World Health Organization (WHO) issued guidelines in 2009 recommending that cART should be initiated for all HIV-infected patients with active TB disease within the first 8 weeks of starting TB treatment regardless of the CD4+ T cell count [12]. Apart from the effects on mortality early initiation of cART in TB-HIV co-infected patients reduces the incidence of tuberculosis across all CD + T cell count levels [13].

The conflicting evidence concerning the optimal timing of cART is based on randomized controlled trials (RCTs) measuring efficacy under ideal, controlled settings that are difficult to replicate in clinical settings [14]. Therefore, an estimate of individual effectiveness will provide a better idea of how TB-HIV co-infected patients with a preserved immunity (CD4+ T count above 350cells/mm^3^) respond to cART under programmatic conditions [15].

We used routinely collected clinical data from the TB and HIV treatment program in the southern part of Zambia to evaluate effectiveness of cART in reducing mortality among TB-HIV co-infected patients with CD4 + T cells above 350cells/mm^3^ at the time of TB diagnosis and TB treatment initiation. We also explored the effect of cART on other programmatic outcomes such as loss to follow up using appropriate statistical methods to address potential bias of the observational program data.

## Methods

### Overview

Using a retrospective cohort study design, we evaluated TB treatment response and survival in 337 HIV-infected TB patients who had CD4+ T cells of 350cells/mm^3^ or greater at the time of TB diagnosis or TB treatment initiation (baseline CD4+ T count).

### Study setting and procedures

The data was collected from the southern province of Zambia between January 2012 and June 2012. The Ministry of Health in Zambia started implementing the WHO guideline of treating all TB HIV co-infected patients at the beginning of 2010 [16]. Between 2006 and 2010 HIV patients with CD4+ T count less than 200 cells/mm^3^ diagnosed with TB were started on cART between 2 and 8 weeks after initiation of TB therapy after being assessed to be clinically stable while on TB treatment [17]. For patients with CD4+ count above 350-cells/mm^3^ cART was differed until completion of TB therapy. In 2010 the guidelines were updated and required that all TB-HIV co-infected patients be initiated on TB therapy followed by cART as soon as possible regardless of the CD4 + T count [12]. However, individual physician practice varied and TB patients were started on cART based on clinical indication as far back as 2006, even with CD4+ T counts above 350cells/mm^3^.

Patient data were abstracted from the TB registers, cART registers and files at Livingstone General Hospital, Monze Mission Hospital, Choma Hospital, Maramba clinic and Mazabuka District Hospital. This analysis included data for both in-patient and out-patient care. The inclusion criteria were: diagnosis of TB by a clinician with or without a sputum smear positive result where full course TB medication was prescribed, HIV seropositive, CD4+ T counts of 350 cells/mm^3^ or greater at time of TB diagnosis (at baseline), and age of 15 years and older. Patients were excluded if they were already on cART at the time of TB diagnosis or had incomplete information available in the medical records. We cross-linked TB clinic with ART clinic records to identify eligible patients.

Medical records were reviewed and data abstracted using standardized data collection forms. The main outcome for this study was all-cause mortality. Death and date of death were ascertained through medical records. When the date of death was not available we used the last date of contact with the clinic as the date evaluated. Patients who were alive at the end of the study period were censored on that date; patients lost to follow-up were censored on the date they were last seen in the clinic or the last drug refill date. The secondary outcomes of the study were completion and cure, default on therapy, and TB treatment failure. Standard definitions according to the National TB Program (NTP) based on WHO guidelines were applied [1].

### Exposure and outcome measures

Our main exposure was initiation of cART during TB treatment. Since this was a retrospective cohort study, the decision to initiate therapy was made by the treating clinician and patient, and not influenced by study investigators. The start date and initial cART regimen were recorded from medical records in the cART clinics. Patient demographic information, clinical and laboratory data were extracted from patient files; the data included stage of HIV disease, Cotrimoxazole prophylaxis, WHO stage of HIV infection, performance status, and hemoglobin.

### Statistical analysis

Observed survival was defined as the time of the initiation of anti-tuberculosis treatment until death or censoring. Separate survival distributions were estimated using Kaplan-Meier methods for patient receiving cART and those not receiving cART; survival distributions were compared using log-rank test. Adjusted Cox proportional hazards models were used to estimate the hazard ratios for death among patients treated with cART versus those not treated with cART. The proportional hazards assumption was tested using graphical methods; no violations of this assumption were found.

Because of the retrospective nature of data collection, we performed a sensitivity analysis to evaluate confounding by indication and the effect of loss to follow-up. To address the confounding by indication, we performed a propensity score analysis. Using a logistic regression analysis, we calculated the expected probability of starting cART for each member of the cohort using a full list of covariates and interactions; this probability was considered the propensity score for starting cART. Using this score, we matched patients who started cART with patients who did not start cART in a one-to-one manner according to the propensity scores. We were able to match 66 pairs of patients (*N* = 132). We then compared the clinical characteristics between the matched pairs to ensure balance. In this propensity matched cohort, we repeated the Kaplan-Meier analysis to estimate covariate adjusted mortality between the groups used an adjusted Cox proportional hazards model to examine the overall effect of cART on survival.

To evaluate the effect of bias due to the loss to follow-up, we evaluated two extreme scenarios. In the first scenario, we assumed that all patients who were lost to follow-up survived until the end of the observation period (August 2012); in the second, we assumed that all individuals lost to follow-up died within 90 days of the date last seen. Survival distributions and hazard ratios for each scenario were compared with the main study results to determine whether study conclusions would be altered in these two scenarios. All statistical analysis was performed with SAS software version 9.4 (Cary, NC, USA) and Kaplan Meier curves were plotted with the survminer package in R-statistical software [18, 19].

## Results

**Fig. 1:**
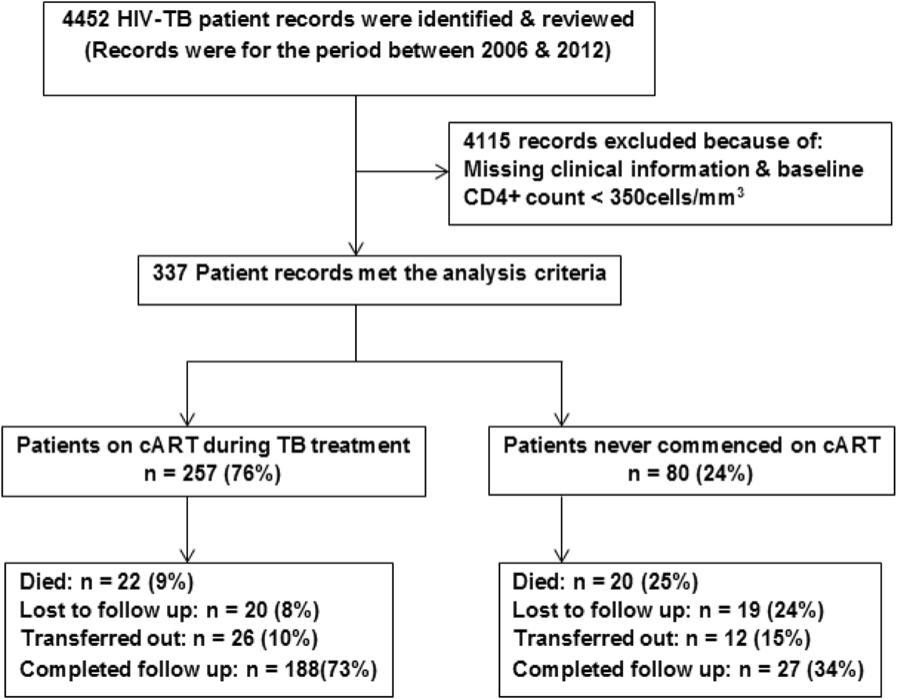
Summary of study enrolment, analysis and outcomes of the 2 cohorts

We identified a total of 4452 patients in the TB registers who were TB and HIV co-infected between January 2006 and August 2012. We cross-linked them with the cART Clinic registers. After applying the inclusion and exclusion criteria, we identified 337 patients who had CD4 + T cell count greater than 350 cells/mm^3^ who were eligible for the analysis; 4015 patients were excluded because of CD4+ T cell count less than 350 cells/mm^3^ and/or had insufficient clinical data (Fig. 1).

Of these 337 patients, 257 (76 %) were started on cART during TB treatment and 80 (24 %) were never started on cART. The patients in the two cohorts were clinically similar in terms of age, sex, hemoglobin level, weight CD4+ T cell count and functional state (Table 1). The median CD4+ T cell count in the patients on cART was 465 cells/mm^3^ (interquartile range: 391, 576) and in patients not on cART, 468 cells/mm^3^ (interquartile range: 397, 611). There were 173 patients on Cotrimoxazole prophylaxis in the cohort on cART (67 %) as compared to 17 for those who were not started on cART (21 %).

**Table 1:** Baseline characteristics among TB/HIV co-infected patients with CD4+ T cell count more than 350cells/mm^3^, for those initiated on cART and those who were not on cART

Clinical characteristic		No cART (*n* = 80)	cART (*n* = 257)
		*n (%)*	*n (%)*
Sex ^a^	Male	41 (51)	106 (41)
	Female	36 (49)	147 (57)
TB Microscopy	Sputum smear positive	23 (29)	43 (17)
	Sputum smear negative	30 (38)	136 (53)
	Extra pulmonary	21 (26)	43 (17)
	Not stated	6 (8)	35 (14)
Functional state	Healthy, able to work	30 (38)	103 (40)
	Ill, able to work	9(22)	43 (17)
	Unable to work	6 (8)	13 (5)
	Unknown	35 (44)	98 (38)
		*Median (Interquartile range)*	
Age, years		35 (28, 39)	36 (30, 44)
CD4+ T cell count, cells/mm3		465 (391, 580)	468 (397, 611)
Hemoglobin, mg/dl		11 (10, 14)	12 (10, 13)
Weight, kg		54 (45, 65)	54 (46, 54)

^a^ Sex was not recorded in 7 participants

The median duration of follow-up was 906 days (interquartile range: 358, 1543) in the cohort on cART and 212 days (interquartile range: 109, 652) in the cohort not on cART. In the cohort of patients on cART, 20 patients (7 · 8 %) were lost to follow-up and 26 patients (10 %) were transferred to another health facility (Fig. 1), whereas among the 80 patients who were never on cART, 19 (24 %) were lost to follow-up and 12 (15 %) transferred care.

**Fig. 2:**
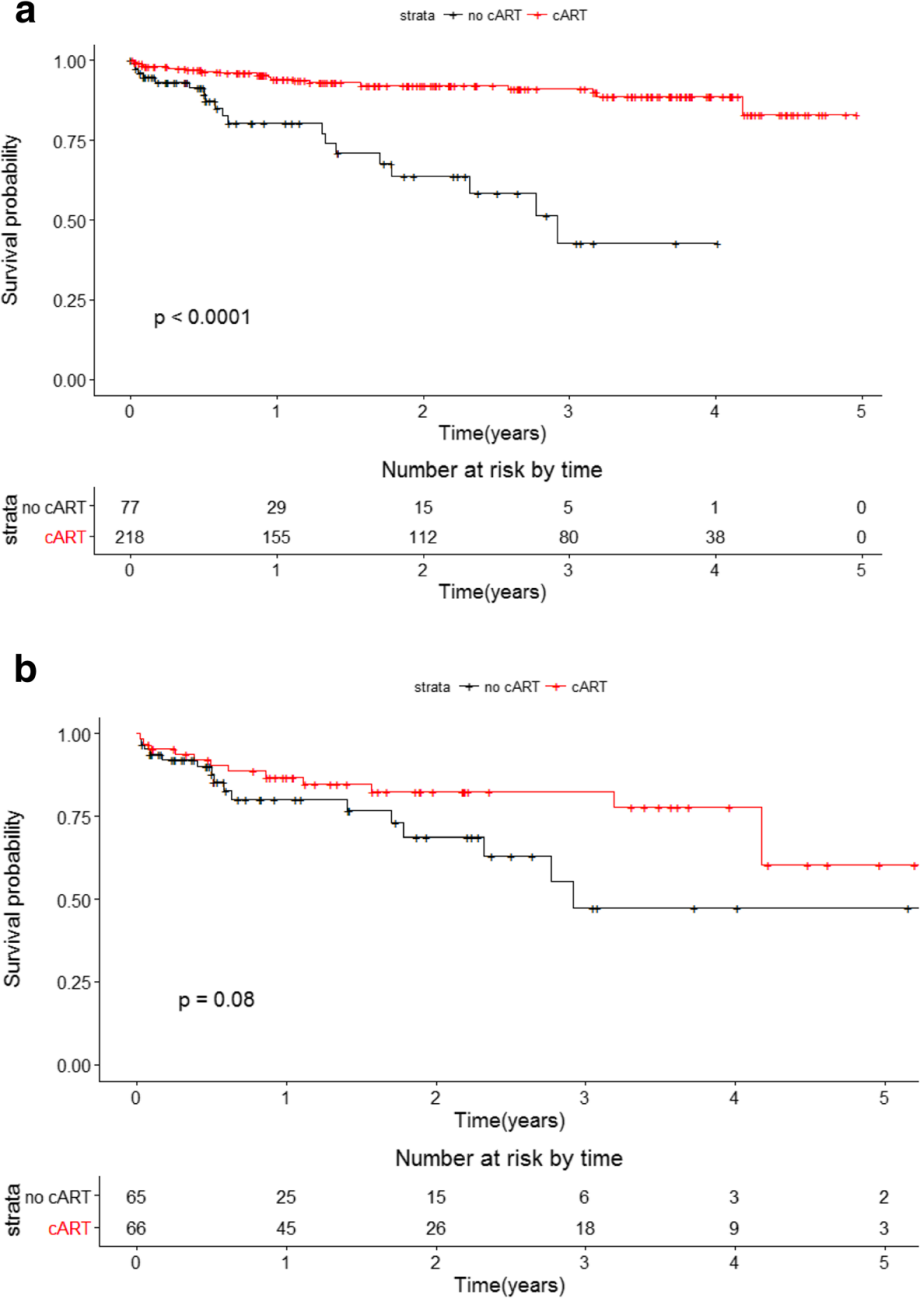
Kaplan Meier survival analysis for entire cohort and propensity score analysis cohort. **a** Entire cohort (cART: n = 257; no cART: n = 80). **b** Propensity score generated cohort (n = 132)

During follow-up, 22 patients died in the cART cohort whereas 20 died in the cohort without cART. Patients on cART experienced a better overall survival compared to patients not on cART (Fig. 2a; *P* value < 0 · 0001, log-rank test). The 1-year survival proportion in patients on cART was 0 · 95 and for patients not on cART was 0 · 81. In a multivariable Cox proportional hazards analysis, the risk of death among patients on cART was reduced by 78 % compared to patients not on cART (adjusted HR = 0 · 22; 95 % CI 0 · 09, 0 · 53), even when controlling for Cotrimoxazole prophylaxis and cure of TB at end of therapy. Of note, Cotrimoxazole use was an independent predictor of survival and reduced the risk of death by 97 % (adjusted HR = 0 · 03; 95 % CI 0 · 01, 0 · 26; Table 2). Survival times used to perform the survival analysis are provided as Additional file 1 (KM_cART.csv).

**Table 2:** Adjusted Hazard ratios for overall cohort and propensity score matched cohort using Cox proportional hazards model

		Overall cohort (*n* = 337)	Propensity score matched cohort ( *n* = 132)
		*………………… Hazard ratio (95 % CI)………………………*	
cART	Yes	0 · 22 (0 · 09, 0 · 52)	0 · 37 (0 · 15, 0 · 93)
	No	1 · 0	1 · 0
Cotrimoxazole	Yes	0 · 03 (0 · 01, 0 · 15)	0 · 09 (0 · 02, 0 · 43)
	No	1 · 0	1 · 0
TB cure	Yes	0 · 34 (0 · 16, 0 · 74)	0 · 28 (0 · 12, 0 · 68)
	No	1 · 0	1 · 0

In a propensity score analysis, 66 pairs of cART treated and untreated patients were closely matched based on the probability of receiving cART. The mean difference in propensity score was -0 · 0045 (standard deviation = 0 · 015), and the distribution of differences was normally distributed. Key prognostic factors for death were balanced between the two groups. Cotrimoxazole treatment was comparable between groups as 24 % of each group received it. When matching for the propensity score, the survival distributions over 4 years differed between the cART treated and untreated cohorts, as in the overall analysis. The 1-year survival proportion in the cART treated group remained better than the cART untreated group 0 · 87 vs. 0 · 80 (Fig. 2b, log-rank test 0 · 08). Early survival during treatment for TB was similar between groups; however, the survival curve separated after about 1 year. In a multivariable Cox regression model of the matched pairs, cART reduced mortality by 63 % (HR = 0 · 37, 95 % CI: 0 · 15, 0 · 93) when adjusting for independent predictors of Cotrimoxazole and TB cure (Table 2).

In a sensitivity analysis to assess the effect of differential loss to follow-up, we found that the results were not altered in the two divergent scenarios. When we assumed that patients lost to follow-up survived until the end of the study, the reduction in mortality was 59 % (adjusted HR = 0 · 41, 95 % CI 0 · 18, 0 · 92; Table 3). When we assumed that patients lost to follow-up died within 90 days of their last recorded clinic visit, the reduction in mortality was 72 % (adjusted HR = 0 · 28, 95 % CI 0 · 16, 0 · 49; Table 3). In both scenarios, the survival distributions between cART treated and untreated patients differed as in the overall analysis.

**Table 3:** Adjusted hazard ratios under two scenarios of follow-up using Cox proportional hazards model

		Full follow-up scenario	Early mortality scenario
		*….Hazard ratio (95 % CI)…*	
cART	Yes	0 · 41 (0 · 18, 0 · 92)	0 · 28 (0 · 16, 0 · 49)
	No	1 · 0	1 · 0
Cotrimoxazole	Yes	0 · 06 (0 · 01, 0 · 26)	0 · 02 (0 · 01, 0 · 07)
	No	1 · 0	1 · 0
TB cure	Yes	0 · 38 (0 · 18, 0 · 84)	0 · 66 (0 · 38, 01 · 13)
	No	1 · 0	1 · 0

Assumptions: *Full follow up scenario*—All patients who were lost to follow-up survived until the end of the observation period (August 2012). *Early Mortality scenario*—individuals lost to follow-up died within 90 days of the date last seen

## Discussion

This study showed that initiation of cART during TB treatment in HIV and TB co-infected patients reduced all-cause mortality by over 60 % among patients with CD4+ T cell counts of more than 350 cells/mm^3^. The effect was durable, lasting up to 4 years. We also found that Cotrimoxazole was an independent predictor of survival and lowered the risk of death by 90 % in these patients, consistent with previous studies [20]. This study is unique because it provides an estimate of individual effectiveness from the long-term survival experience of African patients with HIV-associated TB and relatively preserved immunity. The findings are likely to be applicable to other HIV seropositive patients in other settings in Africa because this analysis is based on program data. The results also reinforce WHO recommendations to initiate cART in TB-HIV co-infected patients concurrently with TB treatment.

Since the advent of antiretroviral therapy in 1996, a central question in HIV care has been when to start treatment [17]. The answer to this question has evolved over time, but today there is convincing evidence from two large, independent studies which show that survival is maximized when cART is started early in the course of HIV infection [5, 6]. As this line of thought evolved, so did the recommendations for cART during TB treatment. All-cause mortality is high during the first year after TB treatment, even when treated with appropriate anti-tuberculosis medications. When cART was added to TB treatment regimens, mortality reduced among patients with advanced immunosuppression [7–11].

On the hand the question of when to start cART during TB has not been conclusively addressed, especially in TB patients with preserved immunity as measured by CD4+ T cell count. In published clinical trials, cART confers a survival benefit among TB patients when started as early as possible during TB treatment among patients with CD4+ T cell counts less than 250cells/mm^3^ [8–10]. However the survival benefit of cART among patients with CD4+ T cells above 350cells/mm^3^ is less certain. Some clinical trials have shown improved survival and improved surrogate markers [7, 21]. A recent multicenter randomized clinical trial in sub-Saharan Africa showed that mortality did not differ between the delayed cART group and the early cART group for HIV co-infected patients with CD4 + T cell count of more than 350cells/mm^3^. This study also showed that mortality was higher at 2 years of treatment for patients on cART [11]. The reasons for these divergent results are unclear.

In our study, Cotrimoxazole prophylaxis was an independent predictor of death and lowered the risk of death by 90 %. The effect of Cotrimoxazole in reducing mortality was consistent with findings of previous studies [22, 23]. Provision of Cotrimoxazole is a minimum standard of care for TB-HIV collaborative activities in Zambia [16]. However patients who were on cART were more likely to be on prophylaxis than those who were not on cART. The difference in Cotrimoxazole prophylaxis coverage may be due to the difference in the quality of care between the two groups.

Our study showed that cART was effective in reducing mortality within the context of an existing health system. The study offers a different perspective on the problem of when to start cART since it is population based and estimates the individual effectiveness of cART as opposed to efficacy, [15] in TB patients with CD4+ T cell count above 350 cells/mm^3^. It also provides a pragmatic answer to the question of whether cART is beneficial in TB patients with higher CD4+ count in the context of clinical care.

Inferences about the benefit of cART in this study are, however, constrained because of confounding by indication and the differential loss to follow-up between those started on cART and those who did not start. As for confounding by indication, this may occur when patients receive treatment based on clinical need, patient preference, or severity of disease. In this analysis, we presumed that patients treated with cART differed in prognosis compared to patients not treated with cART. We adjusted for confounding by indication by performing a propensity score analysis and confirmed the findings of the main analysis. Patients on cART were more likely to be on cotrimoxazole prophylaxis as compared to those who were not initiated on cART. This imbalance may explain survival differences in a crude analysis, but when matching by propensity score, the proportion on cotrimoxazole was balanced between groups and the propensity score analysis confirmed that cotrimoxazole was an independent determinant of survival in these patients and did not attenuate the effect of cART.

As for bias due to loss to follow-up, we observed greater loss to follow-up among the patients not receiving cART. If the reasons for the losses were correlated with death, then there may be a bias in favor of the cART. We suspect a selection bias in this study because in cohort studies from sub-Saharan Africa, a large proportion of those lost to follow-up had died [24]. Since we were unable to ascertain mortality among those who were lost to follow-up, we performed a sensitivity analysis that explored the effect of two extreme scenarios on the results. Regardless of the scenario, the beneficial effect of cART on survival was still evident. Although we cannot estimate the effect of cART precisely using sensitivity analysis, we are confident that the true value of the effect of cART lies between these two extremes.

As for information bias, we did not measure serial CD4+ T cell counts during follow-up, so we are not able to account for the time-dependent effect of the loss of cellular immunity as the CD4+ T cell count declined. If there is a differential decline between treated and untreated patients, then there would be a possible measurement bias. Finally, previous studies have shown that weight and height are drivers of mortality in patients with TB [24, 25]. Although weight was measured regularly in the clinics, height was not, so we were not able to adjust for body mass index in the analysis.

Despite its limitations, our study provides evidence in support of starting cART among HIV seropositive TB patients with preserved immune function, as measured by CD4 + T cell count. It shows the benefit offered by cART and cotrimoxazole in the select group of TB-HIV co-infected patients with preserved immunity.

## Conclusion

In conclusion, starting cART during TB treatment compared to no cART during TB treatment prolongs the survival of HIV-infected TB patients with CD4+ T cell counts above 350cells/mm^3^ in an African setting and is associated with improved retention into care. Our findings support he current recommendations by the WHO to initiate cART in all HIV-infected TB patients regardless of the CD4+ T cell count. Further scale-up of cART during TB treatment is needed to improve health outcomes of HIV seropositive TB patients in Africa.

## Additional file

Additional file 1: Survival times used to perform the survival analysis. (CSV 3 kb).

## Abbreviations

cART: Continuation Antiretroviral therapy
CD4: Cluster Designation class 4
HIV: Human Immunodeficiency Virus
NTP: National Tuberculosis Control Program
NTP: Randomized Control Trial
TB: Tuberculosis
WHO: World Health Organization
